# Galantamine Slows Down Plaque Formation and Behavioral Decline in the 5XFAD Mouse Model of Alzheimer’s Disease

**DOI:** 10.1371/journal.pone.0089454

**Published:** 2014-02-21

**Authors:** Soumee Bhattacharya, Christin Haertel, Alfred Maelicke, Dirk Montag

**Affiliations:** 1 Neurogenetics Special Laboratory, Leibniz Institute for Neurobiology, Magdeburg, Germany; 2 Galantos Pharma GmbH, Nieder-Olm, Germany; University of Leipzig, Germany

## Abstract

The plant alkaloid galantamine is an established symptomatic drug treatment for Alzheimer’s disease (AD), providing temporary cognitive and global relief in human patients. In this study, the 5X Familial Alzheimer’s Disease (5XFAD) mouse model was used to investigate the effect of chronic galantamine treatment on behavior and amyloid β (Aβ) plaque deposition in the mouse brain. Quantification of plaques in untreated 5XFAD mice showed a gender specific phenotype; the plaque density increased steadily reaching saturation in males after 10 months of age, whereas in females the density further increased until after 14 months of age. Moreover, females consistently displayed a higher plaque density in comparison to males of the same age. Chronic oral treatment with galantamine resulted in improved performance in behavioral tests, such as open field and light-dark avoidance, already at mildly affected stages compared to untreated controls. Treated animals of both sexes showed significantly lower plaque density in the brain, i.e., the entorhinal cortex and hippocampus, gliosis being always positively correlated to plaque load. A high dose treatment with a daily uptake of 26 mg/kg body weight was tolerated well and produced significantly larger positive effects than a lower dose treatment (14 mg/kg body weight) in terms of plaque density and behavior. These results strongly support that galantamine, in addition to improving cognitive and behavioral symptoms in AD, may have disease-modifying and neuroprotective properties, as is indicated by delayed Aβ plaque formation and reduced gliosis.

## Introduction

Alzheimer’s disease (AD) is a progressive neurodegenerative disorder and the most common cause of old-age dementia. Neuritic plaques containing amyloid β (Aβ) and neurofibrillary tangles composed of hyperphosphorylated Tau protein constitute major neuropathological hallmarks of AD. The amyloid cascade theory provides a rationale for many features of the disease including the pathological markers, the phenotypes caused by autosomal dominant disease genes, and the risk conferred by the APOE gene status [1]. Increased production of certain Aβ species, their aggregation, and deposition as insoluble plaques is regarded as an early and key pathology in the development of AD [2]. Aβ plaques may serve as reservoirs of soluble Aβ oligomers injuring surrounding neurites and synapses [3], [4]. At a systemic level, therapeutic strategies to reverse or prevent Aβ deposits could lead to partial functional restoration of neural circuits [5]. Therefore, most AD treatment approaches aim at prevention or reversal of Aβ plaque deposition [6], [7].

The acetylcholinesterase inhibitors donepezil, galantamine, and rivastigmine serve as first-line symptomatic drug treatment in mild to moderate Alzheimer’s dementia [8]. Whereas donepezil and rivastigmine are designed acetylcholinesterase inhibitors, the plant alkaloid galantamine additionally acts as an allosterically potentiating ligand of nicotinic receptors, increasing their sensitivity to the neurotransmitter acetylcholine [9]. Chronic low-level stimulation of nicotinic receptors might up-regulate their expression [10], slow down neurodegeneration [11], and confer protection against β-amyloid toxicity [12]. Furthermore, galantamine exerts in cell systems neuroprotective effects by anti-apoptotic action [13], by modulating amyloid precursor processing [14], and by inhibiting β-amyloid aggregation and cytotoxicity [15]. Galantamine activates microglia resulting in enhanced Aβ clearance [16]. Long-term galantamine treatment of AD patients slows down cognitive and global decline [17] and reduces behavioral symptoms, most strongly in patients with moderate or advanced forms of the disease [18]. Similar long-term positive effects are also reflected in PET measurements [19].

The 5X Familial Alzheimer’s Disease (5XFAD) mouse line co-overexpresses APP with three FAD mutations (K670N/M671L, I716V, and V717I) and PS1 with two FAD mutations (M146L and L286V) under the control of the neuron-specific *thy1* promoter [20]. This model recapitulates a variety of AD features, including working memory impairment, reduced anxiety, extensive extracellular plaque formation beginning at 2 months of age, and selective neuron loss, making it a suitable research model for early-onset AD [20]–[24].

In this study, we used the 5XFAD model to investigate the effects of chronic galantamine treatment on behavior and cognition, formation of β amyloid plaques, and gliosis. Our data show that galantamine slows down plaque deposition and improves behavioral performance.

## Materials and Methods

### Ethics Statement

Animal experiments were in line with the guidelines for the welfare of experimental animals and approved by the local authorities of Sachsen-Anhalt/Germany (numbers 42502/2-382 and -945) and carried out in accordance with the European Communities Council Directive of 24th November 1986 (86/609/EEC).

### Mice

5XFAD (B6SJL-Tg(APPSwFlLon,PSEN1*M146L*L286V)6799 Vas/J mice were described by Oakley et al. [20] and were obtained from The Jackson Laboratory (Bar Harbor, stock number 006554). These “5XFAD” transgenic mice overexpress both mutant human APP(695) with the Swedish (K670N, M671L), Florida (I716V), and London (V717I) Familial Alzheimer’s Disease (FAD) mutations and human PS1 harboring two FAD mutations, M146L and L286V. Expression of both transgenes is regulated by neural-specific elements of the mouse *Thy1* promoter to drive overexpression in the brain. 5XFAD transgenic male mice were crossed with B6SJLF1/J female mice (Jackson Laboratory, stock number 100012). The resulting F2-offspring were used in all experiments. Transgenic mice were identified by PCR according to the supplier’s protocol.

### Galantamine

Galantamine hydrobromide was obtained from Macfarlan Smith (Edinburgh, UK). The naturally occurring alkaloid was extracted from daffodil bulbs (Narcissus pseudonarcissus) and was isolated and purified as the hydrobromide salt, as described in the related drug master file. Purity was >99%. The molecular formula is C17H22NO3Br and the molecular weight is 368.28.

### Galantamine Treatment

10 to 12-week-old mice received galantamineHBr dissolved in drinking water at a concentration of either 12 mg/l during four weeks followed by three weeks with 60 mg/l (low dose), or 36 mg/l during four weeks followed by three weeks with 120 mg/l (high dose). Thereafter, the mice were water deprived overnight and received in the morning drinking water containing 120 mg galantamineHBr/l until the behavioral experiments were terminated and animals were sacrificed for histological examination. Behavioral experiments were conducted after eight weeks of treatment and water deprivation was terminated 30 to 60 min before the behavioral test to ensure a high galantamine concentration during the experiment. Water consumption and body weight were monitored during the application period.

### Behavior

For the behavioral analysis, sex- and age-matched littermate wild-type mice were used as controls. During the light phase (12h/12h light-dark cycle), mice were subjected to a series of behavioral tests [25], [26] by an experimenter not aware of the genotype. First, general parameters indicative of the health and neurological state were addressed following the neurobehavioral examination described by Whishaw and colleagues [27] and the tests of the primary screen of the SHIRPA protocol except startle response [28]. Then, the following behavioral paradigms were conducted in sequential order: *Grip strength.* Strength was measured with a high-precision force sensor to evaluate neuromuscular functioning (TSE Systems GmbH, Bad Homburg, Germany). *Rota-rod performance*. Animals received two training sessions (3 h interval) on a rota-rod apparatus (TSE) with increasing speed from 4 to 40 rpm for 5 min. After 4 days, mice were tested at 16, 24, 32, and 40 rpm constant speed. The latency to fall off the rod was measured. *Open field*. Exploration was assessed by placing mice in the middle of a 50×50 cm arena for 15 min. Using the VideoMot 2 system (TSE), tracks were analyzed for path length, visits, walking speed, and relative time spent in the central area (infield), in the area close to the walls (<10 cm, outfield), and in the corners. *O-Maze*. Mice were placed in the center of an open area of an O-maze (San Diego Instruments). Their behavior during 5 min was recorded on videotape. Number of entries into the closed or open areas was counted and the time spent in these compartments was determined using the VideoMot 2 system (TSE). *Light-dark Avoidance*. Anxiety-related behavior was tested by placing mice in a brightly lit compartment (250 lux, 25×25 cm) adjacent to a dark compartment (12.5×25 cm). The number of transitions between the compartments and the time spent within each were analyzed during 10 min. As a test for long-term memory [29], animals were placed at the last day of testing again in the light-dark avoidance box. The latency to enter the dark compartment was measured and compared to the latency at the first time in the box. *Acoustic startle response and prepulse inhibition (PPI)*. A startle stimulus (50 ms, 120 dB) was delivered to the mice in a startle-box system (TSE) with or without preceding prepulse stimulus (30 ms, 100 ms before the startle stimulus) at eight different intensities (73–94 dB, 3 dB increments) on a 70 dB white noise background. After habituation to the box (3 min), 2 startle trials were followed in pseudo-random order by 10 startle trials and 5 trials at each of the prepulse intensities with stochastically varied intertrial intervals (5–30 s). The maximal startle amplitude was measured by a sensor platform.

For conditioned fear testing of 5XFAD mice, the experimental protocol used for the study of Tg2576 mice by Comery *et al.* [30], Jacobsen *et al.* [31], and Schilling et al. [6] was followed closely. Mice were trained and tested on 2 consecutive days. Training consisted of placing the subject in an operant chamber (San Diego Instruments) and allowing exploration for 2 min. Afterwards, an auditory cue was presented for 15 sec followed by a footshock for 2 sec (1.5 mA un-pulsed). This procedure was repeated, and mice were returned to the home cage 30 sec later. 24 hours after training, mice were returned to the same chamber in which training had occurred (context), and freezing behavior (immobility) was recorded. At the end of the 5 min context test, mice were returned to their home cage. One hour later, mice were placed in a novel environment and freezing behavior (immobility) was recorded for 3 min. The auditory cue (CS) was then presented for 3 min and freezing behavior (immobility) was recorded. Freezing scores are expressed as percentage for each portion of the test.

### Histology

Animals were anesthetized with CO_2_ and perfused intracardially with PBS (10ml/min, pH7.4, 10 min) followed by freshly prepared 4% PFA in PBS (10ml/min, 10 minutes). Brains were post fixed in the same fixative at 4°C overnight, serially infiltrated with 0.5M and 1M saccharose for 24–48 hrs each and frozen in methylbutane at around −70°C. 60 consecutive coronal sections (40 µm wide) from approximately bregma 0 to bregma −3.5mm were obtained per animal. In order to normalize regional bias in plaque load, 12 sections 200 µm apart from each other were selected from all animals in every experiment.

#### Nissl staining

Cryosections were subjected to the standard Nissl staining protocol using cresyl violet acetate (Sigma) and viewed using an Axioplan 2 microscope (Zeiss). Images were captured with Photometrix coolSNAP EZ (Visitron systems GmbH) and analyzed with ImageJ software (NIH).

#### Immunofluorescence

Slide-mounted cryostat sections were blocked with BSA (5% in phosphate-buffered saline) for 1 h and then incubated with primary antibody singly or in combination overnight at 4°C in a humidified chamber. Different combinations of primary antibodies used were; 4G8 (Covance, 1/10000) alone for detection of β amyloid, 4G8 together with GFAP (Sigma Aldrich, 1/10000) for detection of reactive astrocytes, and 4G8 with biotinylated Isolectin GSA (Sigma Aldrich, 10 µg/ml) for detection of microglia. Post incubation, the sections were washed with PBS and probed with the secondary antibody/reagents as required, in a sequential manner. Fluorochromated secondary reagents used were Streptavidin AlexaFluor 488 (Molecular Probes, 1/200), Cy3Goat Anti Rabbit (Abcam, 1/200) and Streptavidin Cy3 (Jackson Laboratories, 1 µg/ml). Sections were examined by Axioplan2 (Carl Zeiss) and images captured by Spot RT-KE (Diagnostic Instruments).

#### Immunoperoxidase

Free floating sections were collected, treated with a 1∶1 solution of PBS and methanol with 1% H_2_O_2_, blocked with BSA (5% in phosphate-buffered saline) and incubated overnight at 4°C with 4G8 antibody (Covance, 1/10000) in a humidified chamber. The sections were then treated with Vectashield ABC kit (Vector laboratories) followed by the chromogenic substrate DAB (Vector laboratories). Sections were viewed using Axioplan 2 microscope (Carl Zeiss). Images were captured with Photometrix coolSNAP EZ (Visitron systems GmbH) and analyzed with ImageJ software (NIH).

#### Thioflavin-S staining

An improved thioflavin-S staining protocol [32] was used to ensure reduced photobleaching and tissue damage. Briefly, sections were treated with 0.25% potassium permanganate solution (quenching) at room temperature for 4 minutes followed by 1% sodium borohydride solution for 2–3 minutes. This was followed by incubation with 0.05% thioflavin-S (Sigma Aldrich T1892) solution in 50% ethanol at room temperature for 8 minutes in the dark. Sections were then subjected to 2 washes of 10 seconds each with 80% ethanol, 3 washes of 30 seconds each with water, and post treatment with 5X phosphate buffered saline (pH 7.4) at 4°C in the dark. Sections were mounted in Entellan (Merck) after a brief wash with water and viewed under the FITC filter set of Axioplan2 (Carl Zeiss).

#### Quantitative analysis of plaques and astrocytes

For quantification of plaque load, images were captured by a Spot RT-KE camera (Diagnostic Instruments) at a magnification of 2.5X so as to include the entire hippocampus/entorhinal cortex in a single frame. Plaque load was determined by counting thioflavin-S-positive plaques using ImageJ software (NIH) and Adobe Photoshop (CS3 version). Reactive astrocytes in the hippocampus and the entorhinal cortex were identified using GFAP staining and images were captured by a Spot RT-KE camera at a magnification of 10X. Five to seven sections from each animal were analyzed using ImageJ software (NIH) and GFAP positive astrocytes were counted.

### Statistical Analysis

Behavioral and imaging data were analyzed using analysis of variance (ANOVA with genotype, sex, and treatment as factors) and *post hoc* analysis using Scheffe’s test (Statview Program, SAS Institute Inc., Cary, NC) to determine statistical significance. For the rota-rod, open field, and startle/PPI experiments, statistical analysis was additionally performed using repeated measures ANOVA (with between-subject factor genotype and within-subject factor session). A *P*-value smaller than 0.05 (p<0.05) was considered significant. Regression analysis (Statview Program) was used for the correlation of plaque count and number of reactive astrocytes.

## Results

### Quantitative Analysis of Plaque Deposition in 5XFAD Mice

**Figure 1:**
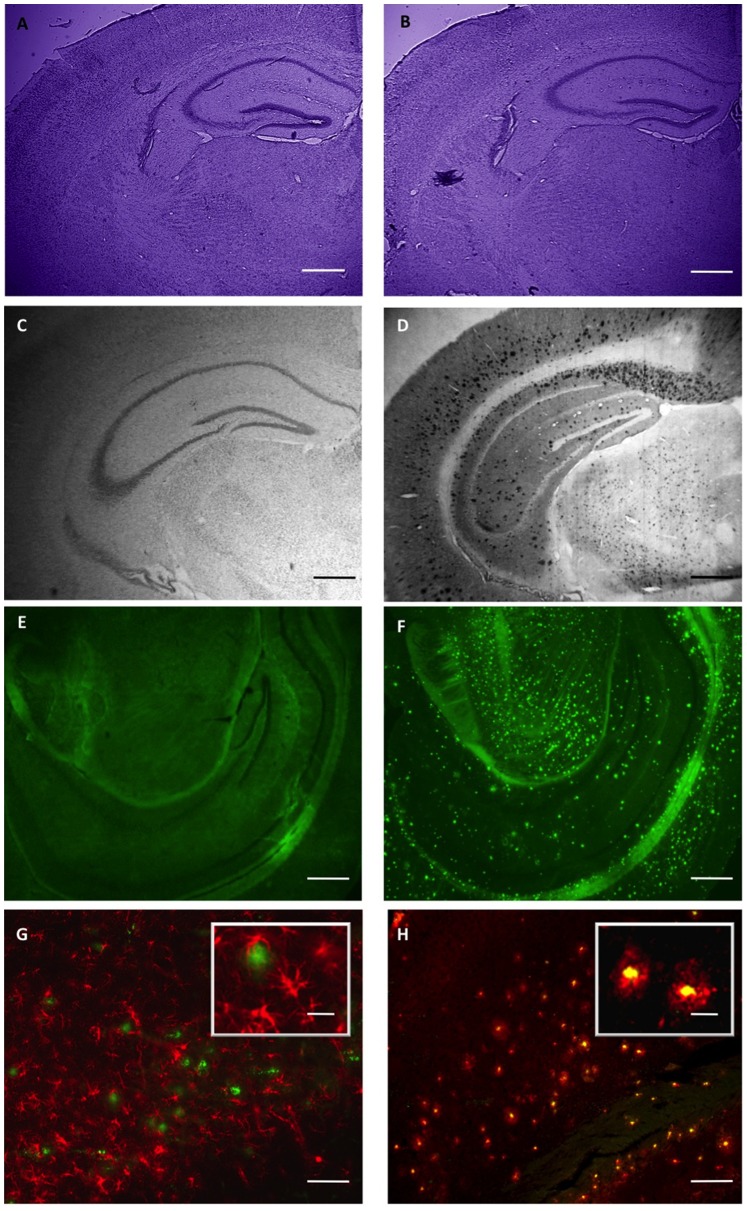
Brain morphology, amyloid plaque formation, gliosis, and microglial activation in 5XFAD transgenic mice. Coronal brain sections from 22-week-old wild-type control littermates (A, C, E) and 5XFAD transgenic mice (B, D, F, G, H) were subjected to Nissl staining (A, B) revealing similar brain morphology. Amyloid β immunohistochemistry (C, D) or thioflavin-S staining (E, F) detect numerous amyloid plaques in 5XFAD mice (D, F), whereas wild-type control brains (C, E) are completely devoid of plaques. GFAP immunohistochemistry (activated astrocytes, red in G) or GSA-lectin (activated microglia, red in H) in combination with thioflavin S staining (green in G and H) revealed plaques surrounded by reactive astrocytes and associated with activated microglia in 5XFAD mice. Scale bars: 500 µm (A–F), 250 µm (G, H), and 100 µm (inset in G and H).

**Figure 2:**
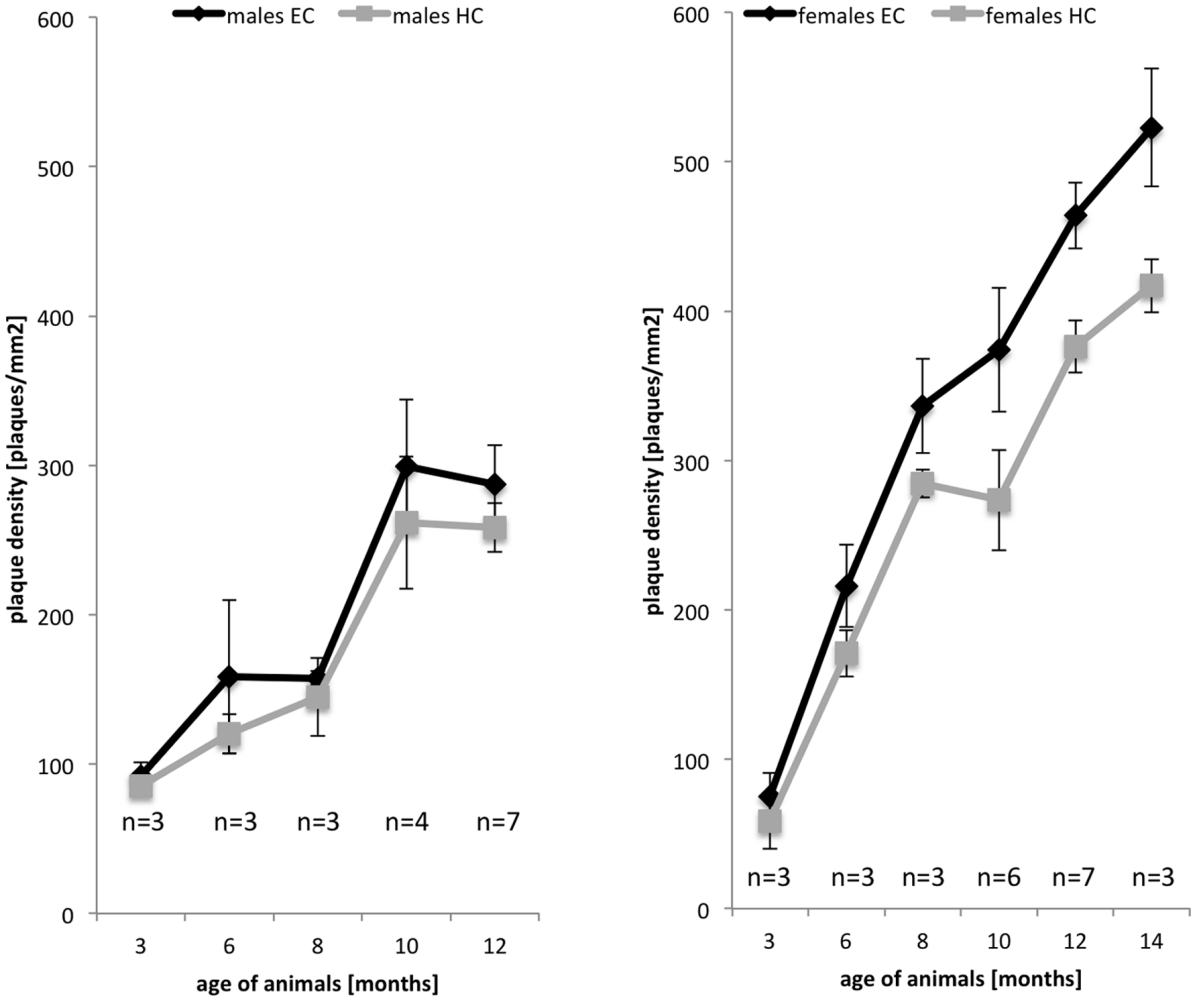
Progression of plaque formation with age in 5XFAD transgenic mice. The plaque density in the hippocampus (HC) and entorhinal cortex (EC) of male and female 5XFAD mice at various ages was determined using thioflavin S staining. In males, plaque density increased in both brain areas and reached saturation at 10 months of age. In female mice, plaque accumulation was faster, reached higher levels, and continued to increase after 14 months of age.

Early deposition of amyloid plaques is a characteristic feature of the 5XFAD mouse model [20]. To exploit this model for the study of drugs for the treatment of AD, we analyzed quantitatively the progression of plaque deposition with age and characterized the behavior at ages with moderate disease progression. In 22-week-old mice, the general brain morphology of 5XFAD transgenic mice is not affected by plaque deposition as exemplified in the hippocampus and cortex by Nissl staining (Figure 1A, B). However, Aβ deposits are easily identified by immunohistochemistry (Figure 1D) or by thioflavin-S staining (Figure 1F). Furthermore, β amyloid deposits are usually associated with reactive astrocytes identified by immunohistochemical staining for GFAP (Figure 1G) and microglia identified by isolectin GSA staining (Figure 1H). Using thioflavin-S as a marker, we investigated the progression of amyloid β deposition with age in two representative brain areas, the hippocampal formation and the entorhinal cortex (Figure 2). Plaques were detectable already in 2-month-old animals. The quantification revealed a strong increase in plaque number and density with age. Maximal density was not reached even at 14 months of age in females, whereas in males no increase in plaque density was observed after 10 months of age. Noteworthy, as also observed for other transgenic Alzheimer’s disease models [33]–[35], we found a strikingly higher number of thioflavin-stained plaques in female compared to male mice. For both sexes, the plaque density was always higher in the entorhinal cortex than in the hippocampus. In control non-transgenic littermates, plaques were never observed at any age. In conclusion, the age before saturation at 10 months appeared to be best suited for the analysis of plaque deposition after a chronic treatment.

### Behavioral Analysis of Untreated Medium Age 5XFAD Mice

**Figure 3:**
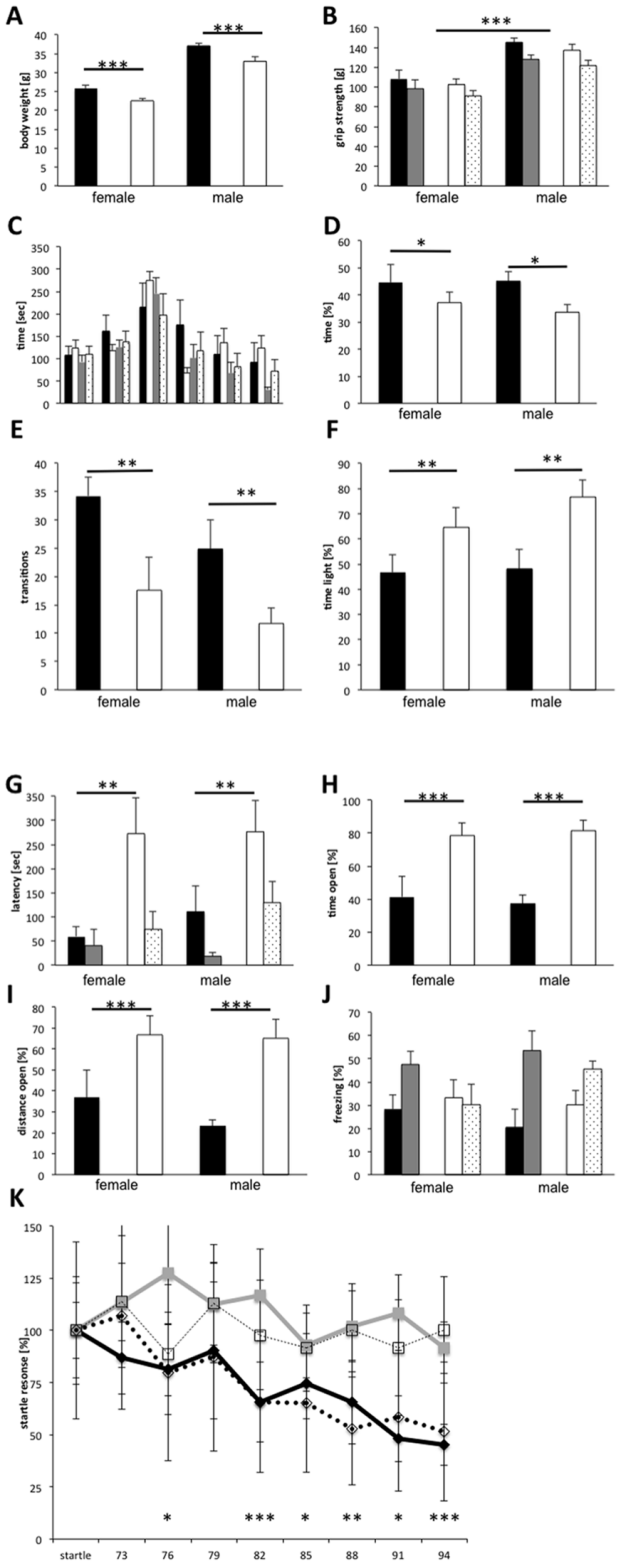
Behavioral analysis of 7-month-old 5XFAD transgenic mice. 5XFAD mice (10 female, 8 male) were analyzed in a series of behavioral tests in comparison to non-transgenic littermates (6 female, 8 male). A significant reduction in body weight (A) of transgenic (white columns) compared to non-transgenic (black columns) mice was observed for both sexes. The grip strength (B) differed between sexes but not between transgenic and non-transgenic mice. Maximum grip strength (black, white columns) and average grip strength (grey, stippled columns) were similar for non-transgenic (black, grey columns) and transgenic mice (white, stippled columns). The rota-rod (C; black control females, grey control males, white transgenic females, dotted transgenic males) did not reveal differences between transgenic and control littermate mice. In the open field (D) transgenic mice (white columns) of both sexes stayed less time in the corners compared to their wild-type littermates (black columns). In the light-dark avoidance paradigm (E, F, G) transgenic mice (white columns) showed less transitions (E), stayed longer time in the light (F), and had a much greater latency to enter the dark compartment (G) both at the first encounter or tested for memory 3 weeks later (grey columns non-transgenic, stippled columns 5XFAD in G). In the O-Maze (H, I) 5XFAD mice (white columns) spend more time (H) and traveled longer distances (I) in the open areas compared to littermate controls (black columns). Fear conditioning (J) did not differ significantly between 5XFAD (white, stippled columns) and control littermate mice (black, grey columns) with respect to freezing in the same context (black, white columns) or when exposed to the tone in a novel environment (grey, stippled columns). Prepulse inhibition of the startle response (K) was not obtained in 5XFAD mice (squares) in contrast to wild-type littermates (diamonds) in both sexes (males open, females filled symbols).

Therefore, we analyzed whether behavioral deficits are detectable in mice with a moderate plaque load. 7-month-old transgenic (10 female, 8 male) and non-transgenic (6 female, 8 male) littermate mice were subjected to a series of behavioral tests. During the neurological examination, transgenic mice did not display obvious abnormalities with respect to body posture, reflexes (uprighting, eye-blink), or general sensory perception (vision, hearing, touch, pain). In contrast, the body weight of transgenic mice was approximately 10% less than that of their control littermates, both, before and after the behavioral tests (Figure 3A) (2-way ANOVA and post hoc analysis, weight before tests: factor genotype F_(1,28)_ = 15.912, p = 0.0004, factor sex F_(1,28)_ = 136,797, p<0.0001; Fisher’s PLSD <0.0001 for each factor; weight after tests factor genotype F_(1,28)_ = 24.426, p<0.0001, factor sex F_(1,28)_ = 147.182, p<0.0001; Fisher’s PLSD <0.0001 for each factor). As expected, the maximum and average grip strength were significantly higher for males compared to females (F_(1,28)_ = 32.038, p<0.0001; F_(1,28)_ = 25.969, p<0.0001, respectively), but did not differ between transgenic and non-transgenic mice (Figure 3B). Furthermore, motoric abilities examined on the Rota-Rod were similar for control and 5XFAD mice (Figure 3C), indicating that motor coordination and motoric capabilities are not generally impaired in 5XFAD transgenic mice of this age.

In the open field, transgenic mice spend significantly less time in the corners (F_(1,28)_ = 5.245, p = 0.0297, post hoc Fisher’s PLSD p = 0.0311) compared to control littermates (Figure 3D). In the light-dark avoidance paradigm, 5XFAD transgenic mice made fewer transitions between compartments (Figure 3E) (F_(1,28)_ = 9.077, p = 0.0054; post hoc Fisher’s PLSD p = 0.0084) and spend more time in the illuminated part (Figure 3F) (F_(1,28)_ = 9.026, p = 0.0056; post hoc Fisher’s PLSD p = 0.0067). In addition, transgenic mice entered the dark compartment after longer latency (Figure 3G, F_(1,28)_ = 8.635, p = 0.0065; post hoc Fisher’s PLSD p = 0.0071). The latency with which transgenic mice entered the dark compartment when tested for memory was still longer as of their non-transgenic littermates (F_(1,28)_ = 4.345, p = 0.0464; post hoc Fisher’s PLSD p = 0.0491). The reduced latency in comparison to the first exposure to the box, however, indicates formation of long-term memories by 5XFAD transgenic mice. In the O-Maze, 5XFAD transgenic mice spent longer time (Figure 3H) and moved longer distances (Figure 3I) in the open areas compared to their control littermates (time F_(1,24)_ = 23,679, p<0.0001; distance F_(1,24)_ = 15.319, p = 0.0007). In summary, 5XFAD transgenic mice spend in these mazes more time in the open illuminated areas.

When analyzed for the startle response and its prepulse inhibition, 5XFAD transgenic mice displayed a significantly reduced startle response at 120 dB (F_(1,28)_ = 4.578, p = 0.0412; post hoc Fisher’s PLSD p = 0.0265), which was not inhibited by prepulses of intensities between 73 and 94 dB (Figure 3K) (repeated measures ANOVA F_(1,196)_ = 11.488, p = 0.0021). Fear conditioning was analyzed using a different cohort of mice. 5XFAD transgenic mice (8 males, 10 females) displayed significantly more freezing in the context (F_(1,32)_ = 4.542, p = 0.0409) but also in the neutral surrounding (not significant, p = 0.1641), thus the context memory (% freezing context - % freezing neutral, Figure 3J) was not significantly different from non-transgenic control littermates (9 males, 9 females). Likewise, tone memory (% freezing with tone in neutral - % freezing neutral without tone) was slightly but not significantly (p = 0.09) less in transgenic mice.

In conclusion, we identified several significant AD related behavioral differences between 5XFAD and control mice already at the age of seven months. Therefore, the 5XFAD transgenic model appears to be a suitable system to study the effects of pharmacologically active substances both on behavior and plaque load.

### Chronic Galantamine Treatment of 5XFAD Mice

**Figure 4:**
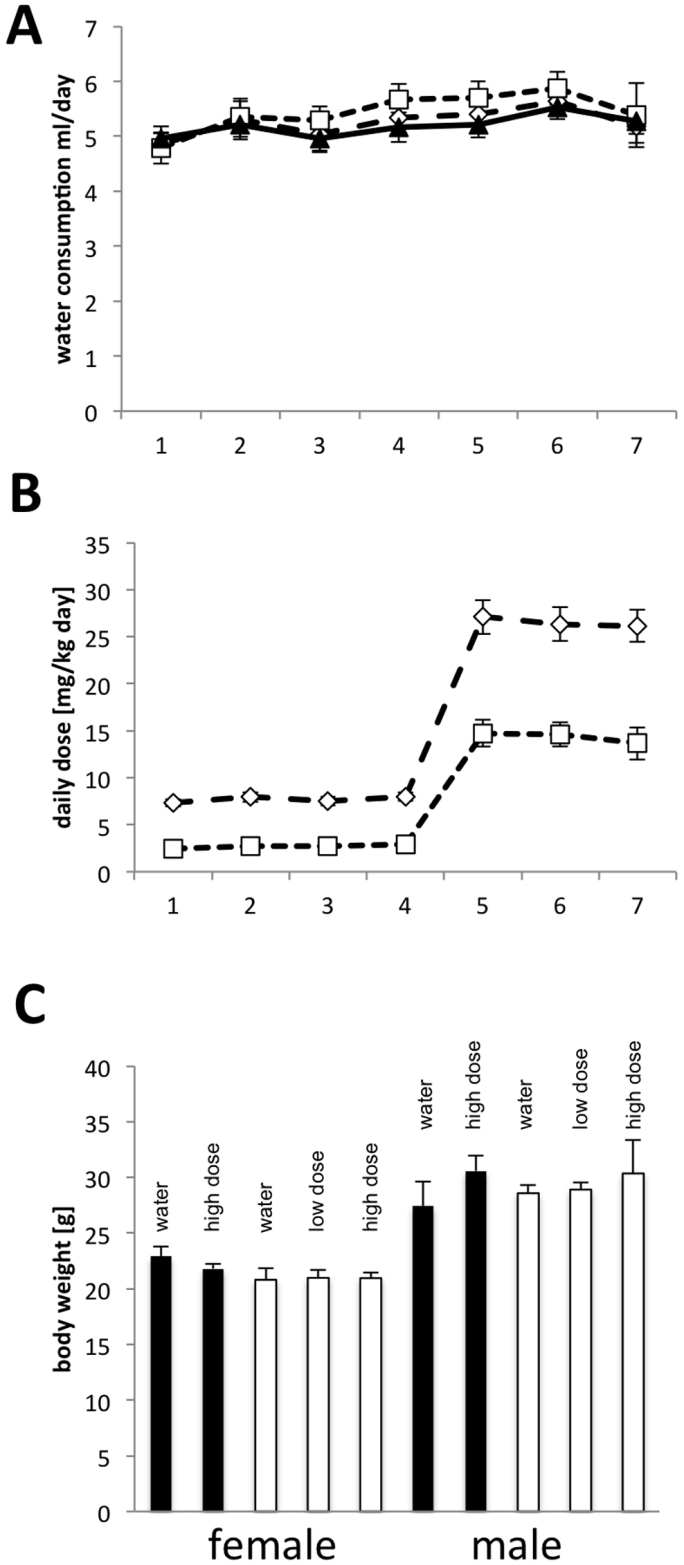
Water consumption, galantamine uptake, and body weight of 5XFAD-transgenic mice treated with galantamine. Water consumption (A) of 5XFAD transgenic was similar irrespective of added galantamine (black triangles: water (n = 16); diamonds: high dose (n = 16), squares: low dose (n = 8). Galantamine uptake (B) was calculated from the amount of drinking water consumed for the low dose (squares, 12 then 60 mg/l) and the high dose (diamonds, 36 then 120 mg/l). The body weight (C) of non-transgenic control (black) or 5XFAD (white) mice was not significantly influenced by the treatment and the behavioral tests.

In order to further explore this model for assessing symptomatic and disease-modifying properties of AD drugs, or drug candidates in development, we investigated the effects of chronic treatment with galantamine on behavior and plaque load. Because this model is characterized by early-onset plaque deposition, we chose to treat 10–12-week-old 5XFAD transgenic mice and non-transgenic littermates with galantamine for 2 months and during the following behavioral tests, which were characterized above for the untreated animals. To reduce any potential adverse side effects, we administered during the first 4 weeks a lower dose of galantamine that thereafter was followed by a much higher dose. During the first 4 weeks, one group of animals received 36 mg/l (high dose) and a second group 12 mg/l (low dose) galantamine in the drinking water. Thereafter, the dose in the first group of animals was increased to 120 mg/l (high dose) and to 60 mg/l (low dose) in the second group. 7 weeks after onset of treatment, the animals were water deprived during the night to ensure a high uptake of drinking water in the morning. During behavioral experiments, animals received 30–60 min prior to testing the drinking solution, so as to ensure a high drug dosage during the experiment. Control animals received water without drug. During the treatment period the general condition, water uptake (Figure 4A), and body weight (Figure 4C) were monitored and did not reveal any difference between treated and control or transgenic and non-transgenic mice indicating that the treatment was well tolerated. This application scheme resulted in the daily uptake of 14 mg/kg body weight (low dose) and 26 mg/kg body weight during the last phase of the treatment (Figure 4B). Consumption of similar amounts of water indicates that galantamine at the concentration used did not induce any preference or avoidance of the drug. Behavioral tests were conducted with 4–5-month-old animals (130–150 days). After the behavioral tests, brains were sectioned for the analysis of plaque density using thioflavin S staining [32], [36].

**Figure 5:**
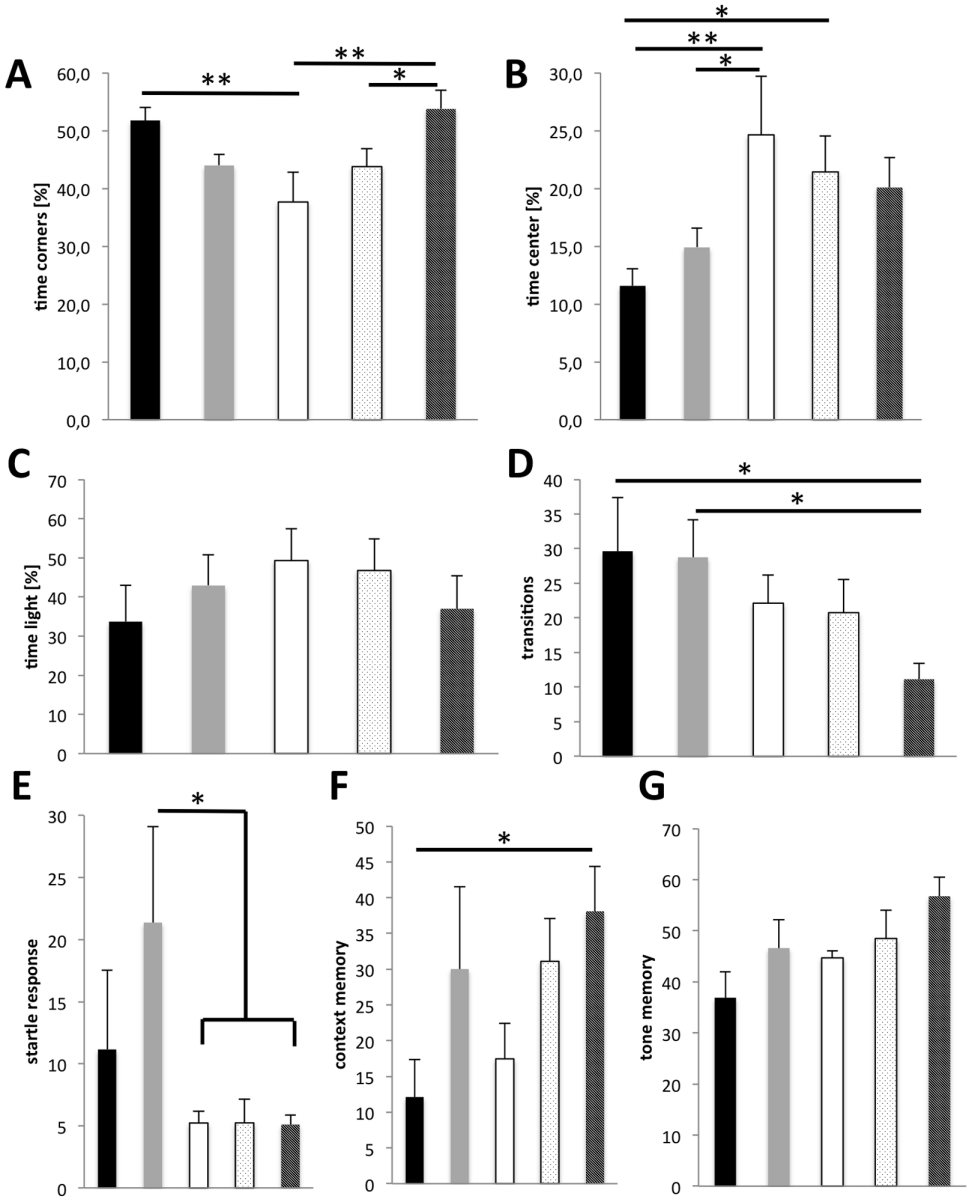
Behavioral analysis of 5XFAD-transgenic mice after treatment with galantamine. In the Open Field (A, B) galantamine treatment restored the preference for the corners (A) and the avoidance of the center (B). Non-transgenic mice receiving water (black columns) showed a significantly higher corner preference (**, p = 0.0045) and avoidance of the center (**, p = 0.0048) compared to untreated 5XFAD transgenic mice (white columns). Treatment of 5XFAD transgenic mice with low dose (stippled columns) or high dose (hatched columns) galantamine increased their corner preference and center avoidance in a dose dependent manner. High dose treated transgenic mice spend significantly (**, p = 0.002) more time in the corners compared to untreated transgenic mice or low dose treated mice (*, p = 0.0451). Non-transgenic mice receiving high dose galantamine (grey columns). In the light-dark avoidance paradigm (C, D), high dose galantamine treatment of 5XFAD transgenic mice (hatched columns) reduced the time in the light (C) to untreated non-transgenic (black columns) control levels. In contrast, the number of transitions (D) is even further reduced by galantamine treatment of 5XFAD transgenic mice (*, p<0.005). The magnitude of the startle response (E) was not affected by galantamine treatment of 5XFAD transgenic mice. During fear conditioning (F, G), galantamine increased the context memory (F) and the tone memory (G) of non-transgenic and 5XFAD transgenic mice similarly.

In the open field, mock-treated transgenic and non-transgenic mice showed similar differences as described above for the 7-month-old animals. Transgenic mice spend significantly less time in the corners of the maze, but treatment with galantamine elevated the preference for the corners to normal levels in a dose dependent manner (Figure 5A). Similarly, reduced avoidance of the center by transgenic mice was restored by galantamine treatment in a dose dependent fashion (Figure 5B). Similar to the results with the older mice described above for the Light-Dark-Avoidance paradigm, mock-treated transgenic mice showed less transitions and longer presence in the illuminated compartment. Treatment with galantamine reduced the time spent in the light to normal levels (Figure 5C), but reduced the number of transitions even more (Figure 5D). The latency of transgenic mice compared to non-transgenic mice was higher on the first exposure and not significantly altered by galantamine treatment (data not shown). The latency at the second encounter with the test box was reduced irrespective of genotype or treatment indicating long-term memory formation. The lower magnitude of the startle response was reproduced with these younger transgenic mice (Figure 5E), but the treatment with galantamine had no effect on the magnitude of the startle response, or its inhibition by prepulses (data not shown). During fear conditioning, freezing in the shock context (Figure 5F) or after the tone in a neutral environment (Figure 5G) by mock-treated transgenic mice was slightly but not significantly higher compared to controls and treatment with galantamine resulted in increased freezing irrespective of the genotype.

In summary, the behavioral differences between transgenic and non-transgenic mice at this early age of 4–5 months confirmed the findings for the 7-month-old mice. The treatment with galantamine improved the behavior in the open field significantly and partially in the light-dark avoidance paradigm but was not able to normalize the startle response. In the fear conditioning paradigm, galantamine generally increased freezing potentially indicating a side effect on anxiety.

**Figure 6:**
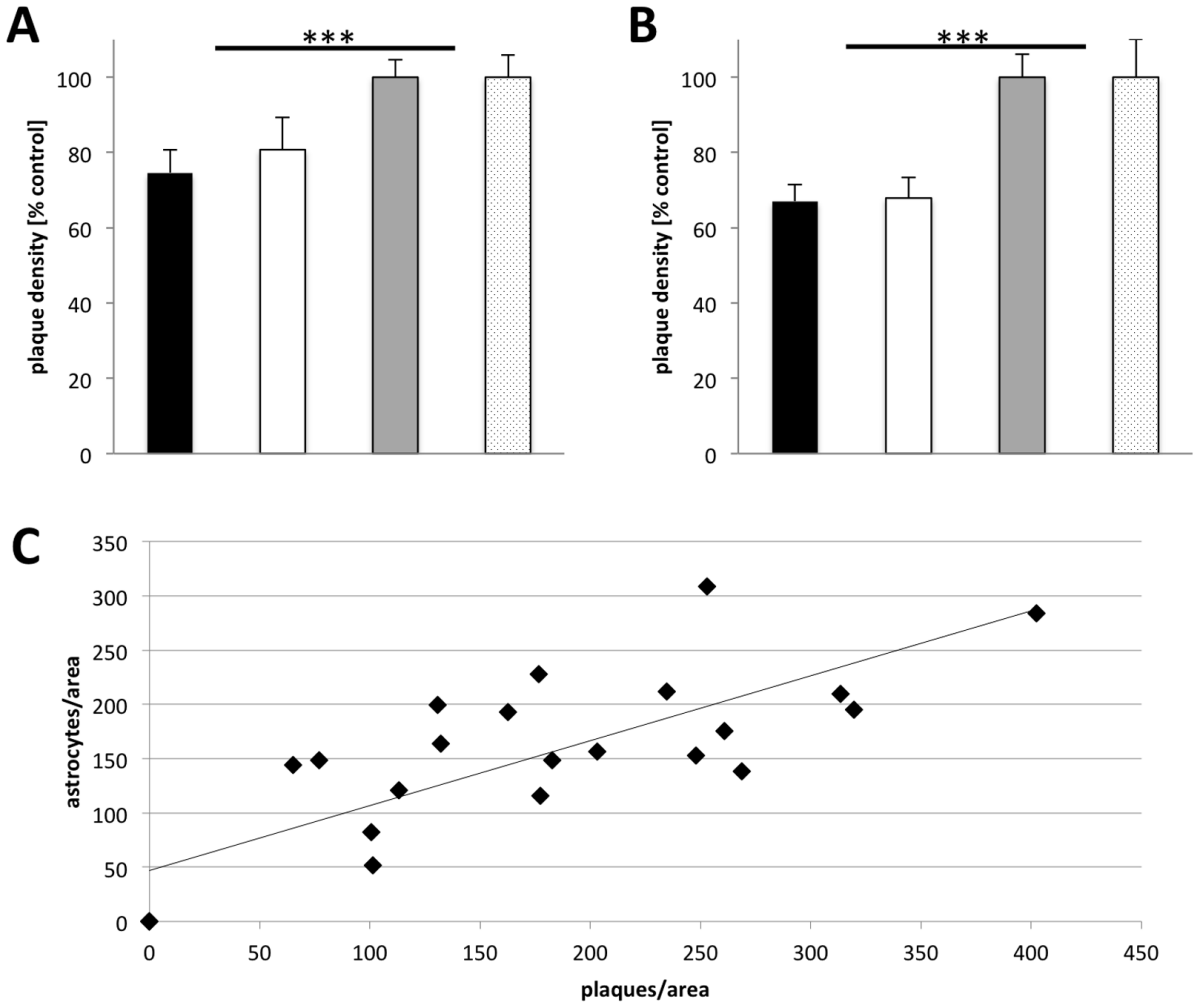
Quantification of plaque density after galantamine treatment. The plaque density in the hippocampus (A) of high dose galantamine treated female (black column, n = 22 sections) and male (white column, n = 47) 5XFAD transgenic mice is significantly reduced in comparison to littermate untreated 5XFAD transgenic mice (female grey column, n = 28; male stippled, n = 50) (***, p<0.0001). The reduction after treatment is even stronger for the entorhinal cortex (B) (treated female black column, n = 27, and male white column, n = 59) (untreated female grey column, n = 45, and male stippled column, n = 59). Astrocyte density and plaque density are strongly correlated in 5XFAD transgenic mice (C) (R^2^ = 0.62; ANOVA F_(1,21)_ = 34.232; p<0.0001).

**Figure 7:**
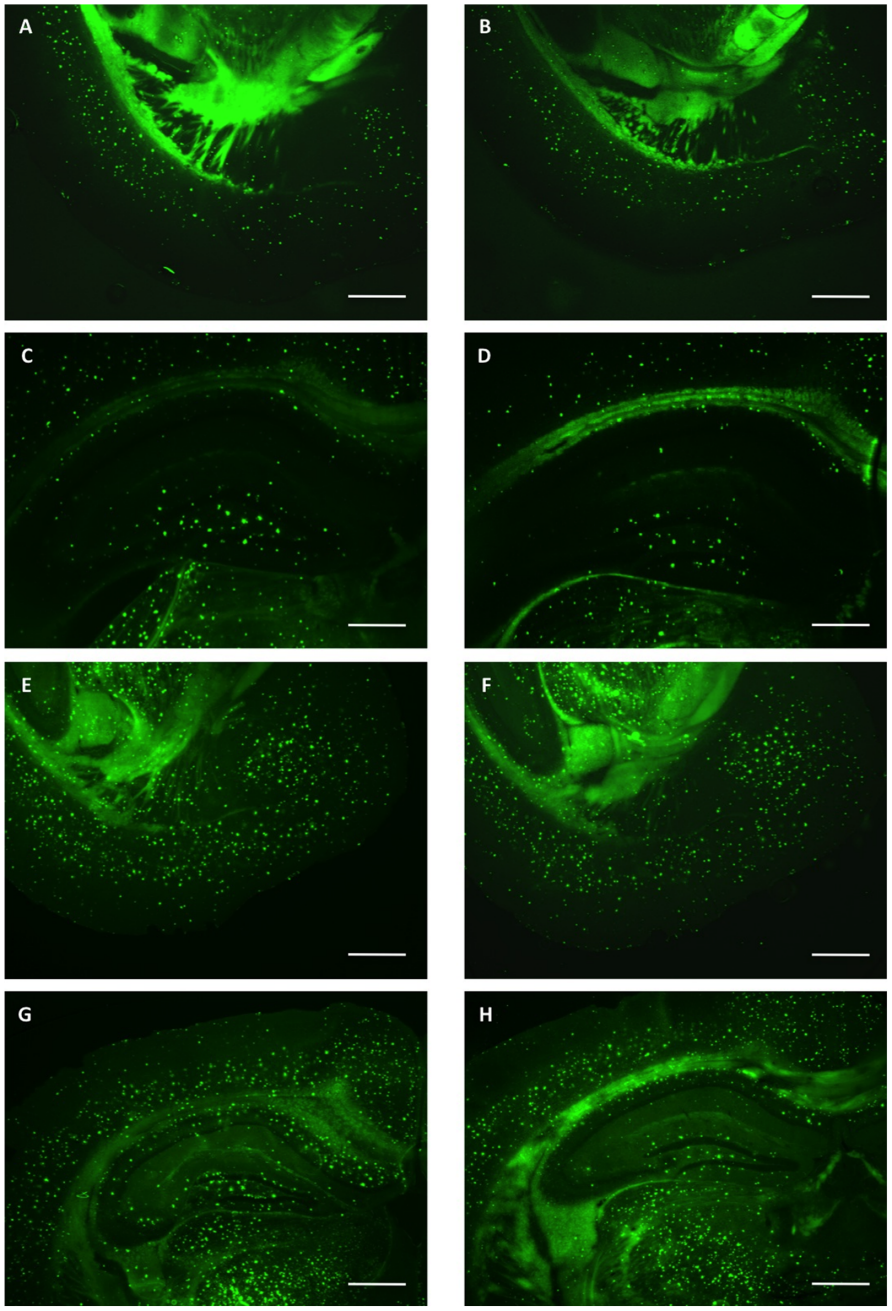
Plaque density after galantamine treatment. Thioflavin S staining of representative coronal brain sections from untreated (A, C, E, G) in comparison to chronically high dose galantamine treated (B, D, F, H) 22-week-old 5XFAD transgenic littermate mice. Fewer plaques are detected in treated animals. Males (A–D) show fewer plaques in comparison to females (E–H), both, in the entorhinal cortex (A, B, E, F) and in the hippocampus (C, D, G, H). Scale bars 500 µm.

Following the behavioral tests, we quantified in the same animals the plaque load in the hippocampus and entorhinal cortex by thioflavin staining. Transgenic animals treated with the high dose of galantamine showed a highly significant lower number of plaques in both areas compared to untreated control mice (p≤0.0001 according to ANOVA) (Figure 6, 7). In the hippocampus of high dose treated transgenic males approximately 19% and in females approximately 25% less plaques were counted compared to untreated controls. In the entorhinal cortex, 32% less plaques for males and 33% less for females were observed. These data indicated that galantamine treatment might reduce the formation of plaques in this model system.

GFAP-positive reactive astrocytes are usually found in association with Aβ plaques (Figure 1). Therefore, we determined the number of GFAP-positive reactive astrocytes in 5XFAD transgenic mice and found a strong correlation between plaque density and the number of reactive astrocytes (Figure 6C).

## Discussion

In this study, the effect of chronic oral administration of galantamine, an acetylcholinesterase inhibitor and allosteric nicotinic receptor modulator, in 5XFAD transgenic mice [20] was investigated.

### Time Course of Plaque Deposition in 5XFAD Mice

The 5XFAD model, due to its early plaque development phenotype, has been investigated extensively to study the various aspects of early onset AD [20]–[24], [37]. However, the pattern of progressive plaque deposition and development has not yet been described in a systematic manner. Here, we monitored for the first time quantitatively the increase in plaque load over time between the age of 3 to 14 months in the hippocampus and the entorhinal cortex, because these plaques are known to closely resemble the amyloid plaques in human AD patients [38]. Moreover, in AD loss of pyramidal cells in lamina 2 of the entorhinal cortex and in the CA1 region of the hippocampus was described [39], indicating that these brain structures are severely affected by the disease. In agreement with previous studies [20], [21], [40], we observed very early onset of plaque deposition in 5XFAD mice beginning around 2 months of age. Furthermore, we show here that plaque deposition occurs differently in the two sexes with lower plaque density reaching plateau levels at 10 months of age in males, while still increasing in females at least until an age of 14 months. A higher plaque density in female transgenic mice has also been noted in other AD mouse models [33]–[35] and is possibly a consequence of decreased estrogen levels [41], modified BACE activity, or altered metal ion levels [42].

### Behavior of Untreated 7-months-old 5XFAD Mice

As therapeutic intervention may be most promising at early stages, we investigated relatively young 5XFAD transgenic mice for behavioral abnormalities. Although, 5XFAD transgenic mice displayed already at the age of seven months a significantly lower body weight paralleling the weight loss always closely associated with AD [43], neuromuscular functioning and motor-coordination appeared normal, confirming previous studies reporting that 5XFAD mice do not exhibit sensory-motoric impairments in string hanging and beam walking before 9 months [24] or abnormal rota-rod coordination before 12 months [22] of age. Likewise, several other AD mouse models display normal motor coordination and grip strength [44]. In contrast, we detected behavioral abnormalities in the anxiety addressing paradigms, namely, open field, light-dark avoidance, and O-maze, similar to the tendency to spend longer times in the center of the open field or the open arms of the elevated plus maze reported previously for 5XFAD [22], [23] and several other AD mouse models [45], [46]. In addition, in the course of the light-dark avoidance test, 5XFAD mice showed a longer latency to enter the dark compartment for the first time. Such behavior could be a consequence of reduced anxiety levels, but in addition, it could also reflect the characteristic aversion to darkness as noticed in AD patients.

Reduced hippocampus dependent trace fear conditioning, but normal hippocampus independent delay fear conditioning, which is comparable to the tone memory assayed here, has been reported for 5XFAD mice [47]. During hippocampus dependent contextual fear conditioning we observed significantly more freezing of 5XFAD mice in the context but also in the neutral surrounding. Therefore, the calculated context memory and the hippocampus independent tone memory were not significantly different. It has also been shown previously, that contextual fear conditioning after 1 footshock is normal in 5XFAD mice younger than 4 months, but impaired in the 6 month old animals, which could be overcome using 3–5 footshocks [48]. Our paradigm used 2 footshocks, which may have been sufficient to overcome a slight deficit.

Suppression of sensorimotor gating has been repeatedly reported in AD patients [49], [50]. In a similar APP/PS1 transgenic mouse model, sensorimotor gating deficits were reported for aged mice using prepulse inhibition of the acoustic startle response [51]. In the 5XFAD mice, we measured a very weak acoustic startle response, indicating possible defects in processing or responding to the stimulus. Furthermore, prepulses of various intensities did not inhibit this residual startle response indicating sensorimotor gating deficits.

In conclusion, 5XFAD mice show at the age of seven months several behavioral deficits, a phenotype with mild cognitive impairment, and progressive amyloid plaque deposition permitting the detection of therapeutic effects.

### Galantamine Treatment Affects Behavior and Plaque Deposition

To investigate the potential effects on the retardation of disease progression, we started with chronic galantamine treatment at 3 months of age, when plaque deposition is considerable, assayed the behavior 2 months later, and then determined the plaque load before reaching saturation levels. Galantamine is known to have side effects with respect to cholinergically mediated gastrointestinal symptoms like other cholinesterase inhibitors [52]. To mimic the treatment regimen in humans, where the dosage is increased with time to minimize negative side effects [53], we increased the dosage gradually during the chronic treatment, and chose 2 different concentrations that were tolerated well by the mice. Also, galantamine did not alter the amount of water consumed, indicating the absence of an aversive response.

The chronic treatment with galantamine had positive effects on certain behavioral tasks but could not rescue all the abnormalities. It did not modify the poor startle response or the lack of its prepulse inhibition in 5XFAD mice. At the moment, we can only speculate that the low startle response results from a very early event that cannot be cured by later treatment or that the dosage or duration were not sufficient to protect these neuronal circuits. Furthermore, the treatment increased the overall freezing behavior in the fear conditioning paradigm irrespective of the genotype, which could be a consequence of the potential “nicotinic effect” of the drug. However, in the open field paradigm galantamine treatment restored the behavior of treated 5XFAD mice to normal. Likewise, the abnormal light-dark avoidance behavior of 5XFAD mice was partially normalized. According to the cholinergic hypothesis, a decreased production of acetylcholine or an amplified acetylcholinesterase activity results in impairment of the cholinergic transmission, in turn leading to the loss of intellectual abilities [54]. Hence, the positive effect of galantamine in restoring certain normal behavioral traits could result from its acetylcholinesterase inhibitor activity.

On analyzing the plaque load in the hippocampal formation and the entorhinal cortex, we found that treated mice show a significantly lower plaque density in both the structures, irrespective of the sex, when compared to the untreated controls. Potential mechanisms of action of the drug may be a reduced deposition of Aβ into plaques as opposed to clearance of already existing deposits. Importantly, acetylcholinesterase has been shown to promote the aggregation of β-amyloid peptide fragments by forming a complex with the growing fibrils [54]. Also, galantamine has been shown to enhance microglial Aβ clearance [16]. Another possible explanation could be that galantamine binds to amyloid oligomers leading to a significant conformational change at the turn region (Asp23-Gly29) disrupting interactions between individual β strands and promoting a nontoxic conformation of Aβ (1–40) to prevent the formation of neurotoxic oligomers [55]. A previous study by Unger et al. [56] reported that galantamine at a concentration of 2mg/kg injected subcutaneously for 10 days had no effect on the levels of soluble or insoluble forms of Aβ in 10 months old Tg2576 mice. In our study, we used a much higher dose, chronic oral application for more than 2 months, and assayed the number of plaque deposits before saturation. Hence, as an extension of this study, quantification of the total Aβ and its different isoforms in chronically treated mice and their relation to plaque density may possibly be helpful to address the mechanisms of galantamine action. However, despite the debate about the most toxic form of Aβ, it is important to note that a number of current strategies e.g. glutaminyl cyclase inhibition and immunotherapy, involved in the treatment and/or prevention of AD progression are indeed directed against the deposition of plaques [6], [57]. Fewer plaques could have a multifold benefit through the reduced levels of all possible harmful forms of Aβ (monomer, oligomer, and fibril). A reduced plaque load has been positively correlated with better performances in behavioral tests [6], [57]. Additionally, regardless of the levels of Aβ, reduced plaque formation could have a role in preventing further cortical atrophy in AD brain, as plaque deposition in multiple studies has been correlated with accelerated cortical atrophy [58], [59]. Furthermore, a reduced plaque burden has been shown to be accompanied by a reduced glial activation [6], [60]. In agreement, we found a positive correlation between the number of plaques and the extent of gliosis, quantified by the number of GFAP positive astrocytes. This implies that chronic galantamine administration reduced the level of astrogliosis in the AD mice brain. Therapeutic targeting of neuroinflammatory pathways in which astrocytes have a prominent position may be a promising strategy to cure AD [61]. The potential of galantamine to interfere with the extent of gliosis is, therefore, another positive outcome of the treatment.

In summary, we report a significantly delayed progression of amyloid plaque deposition and improvement of certain behavioral symptoms associated with AD in the 5XFAD Alzheimer’s disease model after chronic treatment with galantamine.

In contrast to other cholinesterase inhibitors e.g. donepezil and rivastigmine, the relatively hydrophilic galantamine poorly penetrates the blood brain barrier. However, the much more hydrophobic Gln-1062 (Memogain), a pro-drug of galantamine, possesses a more than 15-fold higher bioavailability in the brain [62]. Therefore, it will be interesting to investigate Memogain in comparison to galantamine in this AD model in the future.
